# Semantic Assembly and Annotation of Draft RNAseq Transcripts without a Reference Genome

**DOI:** 10.1371/journal.pone.0138006

**Published:** 2015-09-22

**Authors:** Andrey Ptitsyn, Ramzi Temanni, Christelle Bouchard, Peter A. V. Anderson

**Affiliations:** 1 Sidra Medical and Research Center, P.O. Box, 26999, Doha, Qatar; 2 University of South Florida Sarasota-Manatee, 8350 N. Tamiami Trail, Sarasota, FL, 34243, United States of America; 3 Whitney Laboratory for Marine Biosciences, University of Florida; 9505 Ocean Shore Blvd, Saint Augustine, FL, 32080, United States of America; 4 Department of Physiology and Functional Genomics, University of Florida; 9505 Ocean Shore Blvd, Saint Augustine, FL, 32080, United States of America; University of North Carolina at Charlotte, UNITED STATES

## Abstract

Transcriptomes are one of the first sources of high-throughput genomic data that have benefitted from the introduction of Next-Gen Sequencing. As sequencing technology becomes more accessible, transcriptome sequencing is applicable to multiple organisms for which genome sequences are unavailable. Currently all methods for *de novo* assembly are based on the concept of matching the nucleotide context overlapping between short fragments-reads. However, even short reads may still contain biologically relevant information which can be used as hints in guiding the assembly process. We propose a computational workflow for the reconstruction and functional annotation of expressed gene transcripts that does not require a reference genome sequence and can be tolerant to low coverage, high error rates and other issues that often lead to poor results of *de novo* assembly in studies of non-model organisms. We start with either raw sequences or the output of a context-based *de novo* transcriptome assembly. Instead of mapping reads to a reference genome or creating a completely unsupervised clustering of reads, we assemble the unknown transcriptome using nearest homologs from a public database as seeds. We consider even distant relations, indirectly linking protein-coding fragments to entire gene families in multiple distantly related genomes. The intended application of the proposed method is an additional step of semantic (based on relations between protein-coding fragments) scaffolding following traditional (i.e. based on sequence overlap) *de novo* assembly. The method we developed was effective in analysis of the jellyfish *Cyanea capillata* transcriptome and may be applicable in other studies of gene expression in species lacking a high quality reference genome sequence. Our algorithms are implemented in C and designed for parallel computation using a high-performance computer. The software is available free of charge via an open source license.

## Introduction

Transcriptome sequencing is arguably the first truly high-throughput technology, allowing for the creation of large-scale genomic databases. Expressed sequence tag (EST) libraries are relatively easy to produce and sequence. With proper analysis such projects can give a coarse-grain snapshot of gene activity in a particular sample. In the absence of fully sequenced genomes, transcriptome sequencing remains a good approximation to ascertain the genes present and expressed in a particular organism or tissue, often setting the stage for genome sequencing projects [1, 2]. Recent advances in Next-Generation Sequencing technology (NGS) have increased the utility of transcriptome sequencing by providing better coverage. NGS transcriptome studies also allow quantitative estimation of gene expression by counting the number of reads aligned to each transcript or gene sequence. However, analysis of a transcriptome presents a significant challenge due to the volume and high fragmentation of data, especially in the absence of the reference genome. Among the organisms serving as models for biomedical research, only a relative few have a complete genome sequence available in public databases. As such, transcriptome sequencing remains one of the best options for the analysis of gene expression in non-genomic model organisms. This study was motivated by the challenge of analyzing a MiSeq (Illumina Inc., San Diego) project on the mRNA of the peri-rhopalial tissue of jellyfish *Cyanea capillata*, an important model for analysis of the molecular mechanisms of cellular excitability and evolution of the nervous system.

*Cyanea capillata* (Phylum *Cnidaria*; Class *Scyphozoa*) is a large jellyfish that thrives in the coastal waters of the Northern hemisphere [3]. Cnidarians occupy a basal position within the phylogeny of metazoans and are, most importantly, one of the earliest phyla to possess a nervous system.

Rhopalia are complex sensory bodies located at the margin of the bell [4]. They serve to generate the animal’s swimming rhythm and are connected to the animal’s swimming muscles by way of a diffuse nerve net composed of relatively large, randomly oriented bipolar neurons—the motor nerve net (MNN). When exposed on the acellular mesoglea [5], the MNN provides a remarkably good preparation for studying synaptic [6] and axonal physiology [7, 8]. Interestingly, the latter work [7, 8] has shown that MNN neurons and their synapses share many morphological and physiological characteristics with vertebrate neurons. One goal of on-going research is to identify the neurotransmitter(s) at the fast chemical synapses that connect MNN neurons. The transcriptome of MNN neurons will provide useful information about the variety of neurotransmitter receptors and the uptake mechanisms present in these neurons, thereby aiding the search for neurotransmitters. This neuronal transcriptome will also provide important information about the genetic definition of a neuron, and further our understanding of neuronal diversity and nervous system evolution.

This analysis of the transcriptome of the peri-rhopalial tissue was performed in preparation for an analysis of the transcriptome of isolated MNN neurons contained therein. Cell types present in the peri-rhopalial tissue include MNN and other neurons, epitheliomuscular cells, striated muscle, gland cells and the occasional cnidocyte (sting cell).

There are many software solutions available for the analysis of NGS transcriptome data [9]. Some assemblers, such as SOAPdenovo-Trans [10], Oases [11] and Trinity [12] are designed for unsupervised clustering of RNAseq reads. These tools differ in details and implementation, but operate on the same principle of step-wise integration of fragments based first on nucleotide context overlap, then on end pairing information. In instances of low coverage, high polymorphism and poor RNA quality, the overlap and matching pair information alone might not be sufficient to assemble a representative number of transcripts. Unfortunately, these three aggravations are quite common in marine genomics, which often involves rare and exotic animals, non-canonical model organisms, low RNA yields, poor sample quality and limited funding—challenges that are compounded by the sheer number and diversity of species to target. The idea of using additional information about protein sequences to stitch together transcript fragments lacking overlapping nucleotide sequence has been proposed by Surget-Groba and Montoya-Burgos [13]. This method, however, requires proteome data from closely related species, which is also not available for many taxons. Here we present our own solution for transcriptome assembly that extends existing software with new programs for filling the gaps or adding functionality to the analysis workflow. Our solution exploits biologically relevant information accumulated in the entire sequence databases (such as SwissProt) to brace transcriptome fragments in cases where raw read sequence context alone is not sufficient and a single closely-related protein may also not be present in the database.

## Results and Discussion

Condensing spare reads into draft contigs is a common task for which multiple software solutions are available. For the case study of the *C*. *capillata* peri-rhopalial tissue transcriptome our primary objective was to identify expressed genes and make a reasonable guess about the function of these genes. Multiple studies have established the utility of the RNAseq approach for quantitative estimation of gene expression [14]. A single snapshot of a transcriptome means it would be impossible to be precise, but some quantitative information is still present in the data. For a secondary objective we would like to estimate which of the identified genes are highly expressed and which are poorly expressed, with all possible intermediate values. However, there is still a gap between the end of the read assembly pipeline and the answers to specific questions relevant to the biology of the organism being studied. Some software packages (e.g. Oases [11], the transcriptome assembly version of the Velvet package [15]) propose a two-step strategy: first, the reads are assembled, then original reads are mapped back to draft contigs and scaffolds using third-party software (Bowtie [16]), before matches are tallied and quantified into expression estimations by independent software such as eXpress [17] (http://bio.math.berkeley.edu/eXpress/).

In our experience this multi-step solution led to an unacceptable loss of reads: only about 2,000 draft transcripts could be assigned any estimated expression value, despite the fact that all transcripts were assembled from the same pool of reads. There may be different explanations for why so many contigs, derived from two or more raw reads by one program, could not be matched back to any original raw reads by another program. It would appear that these independent developers all employ different algorithms in their software. Searching for a combination of parameters that can produce coordinated results with independent software tools and keep working through quickly-changing versions would not be a viable solution.

Conveniently, the SOAPdenovo [18] assembler stores all original matches of reads forming contigs in one of the intermediate files. However, direct application of SOAPdenovo assembler is also problematic: the output contains 321,269 contigs and scaffolds, which is far beyond all reasonable expectations for the number of expressed genes in a jellyfish transcriptome. Here we propose a solution that creates a reasonable qualitative (in terms of assembles and annotated transcripts) and quantitative (in terms of relative abundance and total number of expressed transcripts) reconstruction of the transcriptome, based only on short NGS reads.

**Fig 1:**
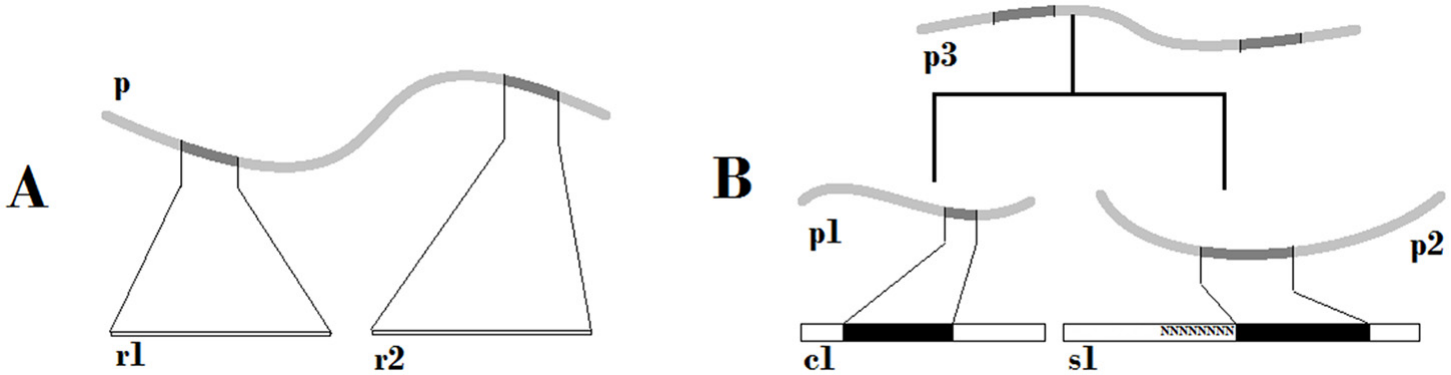
Joining draft assembly fragments using database protein sequence. **A**. The simplest case of two singleton reads, r1 and r2, which have no overlapping sequence. Both reads encode fragments of the same protein from a different species found in the database. **B**. Example of a more complex relationship between a non-overlapping (contig c1) and a scaffold (s1) from the draft assembly. They may encode parts of the same protein. However, if there is no single closely-related protein in the database the nearest homolog p1 for c1 belongs to one species and the s1 encodes protein fragment most similar to p2 in a different species. Since p1 and p2 are proteins homologous to each other this information can be used to brace fragments c1 and s1 of unknown genome.

The central idea of our approach is to bring more biologically-relevant information into the process of read assembly. In the case of traditional *de novo* assembly, the first mechanistic step of the process, reads are put together on the basis of significant overlap in the primary sequence. Both raw reads and partially assembled fragments are sufficient to generate hypothetical protein fragments using 6-frame translation. If two or more of such fragments are homologous to a known protein sequence with a high confidence it is reasonable to cluster such fragments together. The principle is illustrated on Fig 1. Even if two fragments have no overlap in the primary nucleotide sequence they can be identified as coding parts of the same protein. Protein sequences, even from other distantly-related species, can brace together fragmented transcripts. All fragments clustered together contribute pieces of functional annotation to the characteristics of the resulting supercontig. Likewise, all fragments braced by a protein sequence contribute their read count to the final estimation of quantitative level of expression. The down side of this approach is that transcripts originating from closely related paralogous genes could not be separated. It is possible that some of the clusters we report are made of two or more closely related genes. However, we believe this tradeoff is reasonable for a first approximation at the composition and function of an unknown genome.

We have implemented the software that generates annotated clusters from the standard tabulated output of a BLAST [19] search of draft contigs and scaffolds in a databank of known proteins. The only input file is the standard tabulated output of the BLAST search. The parallel version has additional parameters specifying the number of threads to employ for clustering. A separate program was developed to collect the originally-detected matches of reads forming draft contigs, match them to corresponding scaffolds and then to super-scaffolds (clusters of draft contigs and scaffolds). The program can extract read matches from the intermediate output of the SOAPdenovo program (not all assemblers store this information) and produce a crude estimate of transcript abundance. The output is the table (tab-delimited text) of super-contigs, the nearest homologs (estimated by lowest BLAST e-value) in the protein database.

Parts of the workflow are flexible and can be substituted by similar software solutions. For instance, SOAPdenovo can be substituted by a different NGS assembler (such as Velvet, Newbler, or ABySS) if there is a reason to believe a particular program is producing better assembly of a particular set of reads. In this case one would have to skip the quantitative estimation of expression intensity, or add an additional program converting the format of read match information for input. The BLAST search can also be substituted by BLAT, HMMer or other similarity detection software, so long as the output format can be converted to BLAST-style tabulated form. We used the Swissprot database to find the nearest annotated homologs, but there is a wide choice of standard and custom data sets that can be used in the modified workflow. For example, using a non-redundant database is likely to result in more super-contigs discovered and a better representation of the unknown transcriptome. If phylogenetic analysis is the main objective of the subsequent analysis, OrthoMCL database [20] could be a good choice. For this case study we decided in favor of the smaller and better annotated Swissprot database, since the addition of super-contigs that do not match any previously characterized proteins increases the computational load for no net gain in our knowledge of jellyfish gene expression, or the goals of the study.

**Fig 2:**
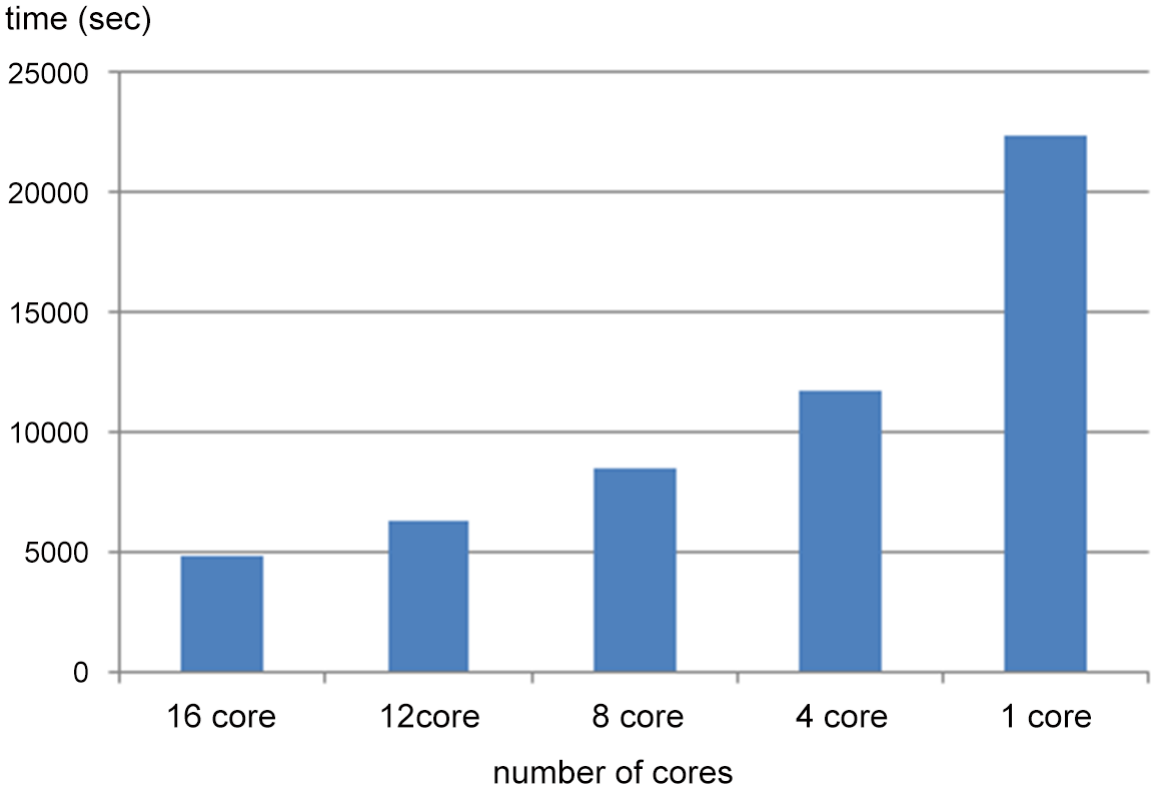
Benchmarking the parallel version of the draft transcript clustering program. The ordinate shows seconds elapsed per clustering iteration while running the same application on a single NGS read library with different number of threads allocated on a shared 6-CPU 24-core Linux machine.

Clustering is the most computationally demanding part of our workflow. The single-CPU version took more than three days to complete the clustering of our data set (which is a typical Illumina MiSeq transcriptome sequencing project). The other computationally-demanding programs we used in the workflow (SOAPdenovo assembler and BLAST database search) are already designed for parallel computation. Our scalable parallel version of super-contig clustering uses POSIX standard threads. We chose this standard because it is relatively easy to optimize and debug, portable to most common platforms and computationally effective. The clustering algorithm we applied is also easy to implement in parallel threads, which makes a sophisticated message-passing interface (MPI) unnecessary. Scalability was tested on the University of Florida High-Performance Computing Center (HPC) testing and development node with 24 cores (4-CPU Opteron 8435, 2.6GHz and 64GB RAM). The diagram of test results is presented in Fig 2. Overall performance scales reasonably well up to 16 cores available to run on a shared computer. All threads start simultaneously and were assigned equal shares of data to process. However, different threads may require up to twice the time to complete calculations due to random differences in data and interference by other processes competing for core allocation. This effect became more apparent when more processing cores were allocated. Overall time to completion is two to three days, depending on the choice of parameters and the number of computing cores available. SOAP assembly runs overnight (10–12 hours; Trinity alternative takes about twice the time to complete assembly of the same data). Super-scaffolding also works overnight, but can be accelerated by allocation of additional cores. In the process of computation the performance monitor indicates 100% CPU load for super-scaffolding, ~74% for BLAST against Swissprot and about 10% for Trinity assembly.

**Fig 3:**
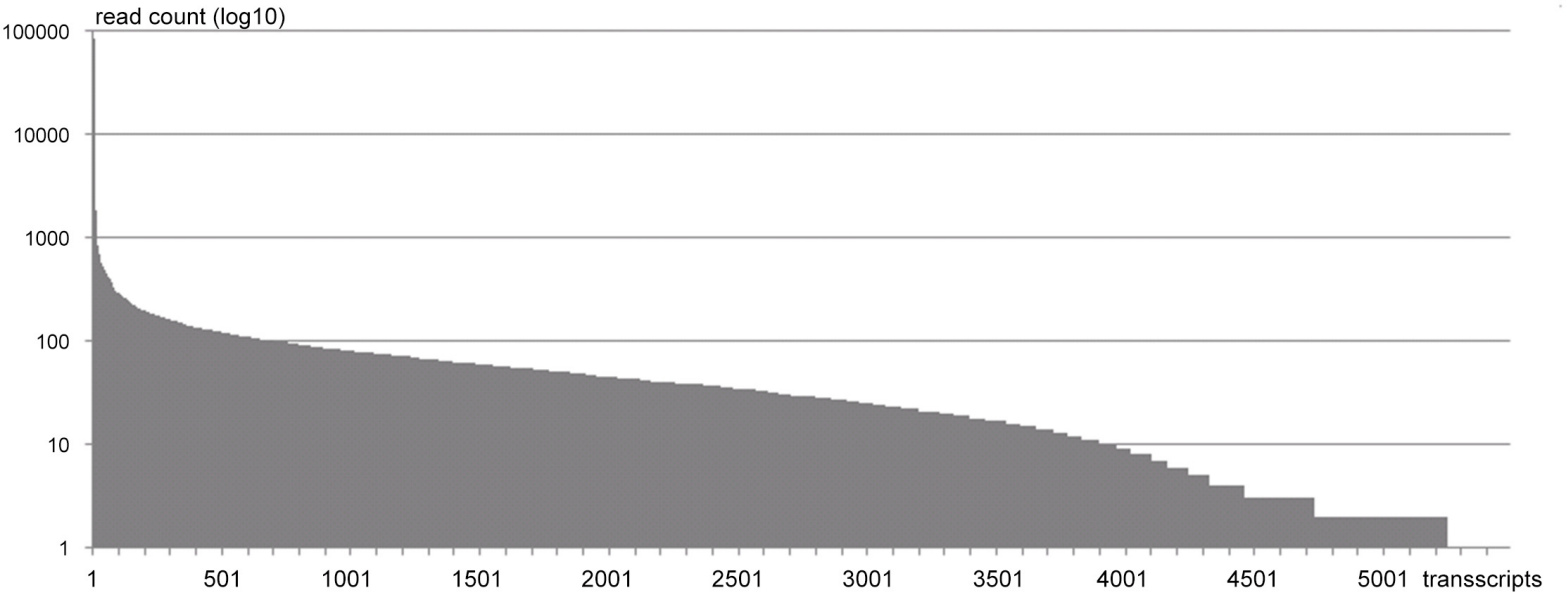
Distribution of resulting read counts (vertical axis, log10 scale) per transcript (horizontal axis) estimating expression intensity.

The case study data set sequenced using Illumina MiSeq contained 8,557,893 paired-end reads, 150 b.p. each. The preliminary sequence overlap assembly by SOAPdenovo produced 321,269 fragments of length longer than original reads. The BLAST search produced a table (tab-delimited text) containing 1,867,307 hits, i.e. graph edges with fidelity above certain threshold (e = 0.001). The threshold was selected to eliminate BLAST hits resulting from low complexity or coincidence of functional motifs. This parameter can be corrected by the user if applied to a different dataset. A quick filter removed duplicated edges, leaving 1,112,212 for clustering. A duplicated edge in this context is a line in BLAST tabulated output that lists the same query and the same database homologous sequence IDs as some other line (i.e. pointing to different homologous areas in the same pair of sequences). Clustering algorithms required 8 iterations to produce the final list of 3,512 non-redundant transcript clusters ranging from 1 to 764,037 members (edges). The output table also lists the nearest homologs to each cluster. The list of homologs from the output table is then submitted to the input of DAVID Bioinformatics Resource as a list of genes for functional annotation and functional classification. We used the standard web interface. Automation through recently released API (http://david.abcc.ncifcrf.gov/content.jsp?file=DAVID_API.html) could be a good option for routine processing of multiple data sets in the future. Partial results of the functional classification are included in the supplemental materials (S1 Table). The overview of the resulting distribution of read occurrence (estimation of transcript abundance) is given in Fig 3. Overall we can claim good progress from over 300,000 fragments to a manageable number of draft transcripts of the same order as expected number of genes actively expressed in the sample of jellyfish tissue. The dynamic range of gene expression is also on the same order as we used to see in microarray experiments, between 1 and 83,000 copies, which allows approximate placement of all identified and annotated genes into highly expressed, low expressed and intermediate categories.

**Fig 4:**
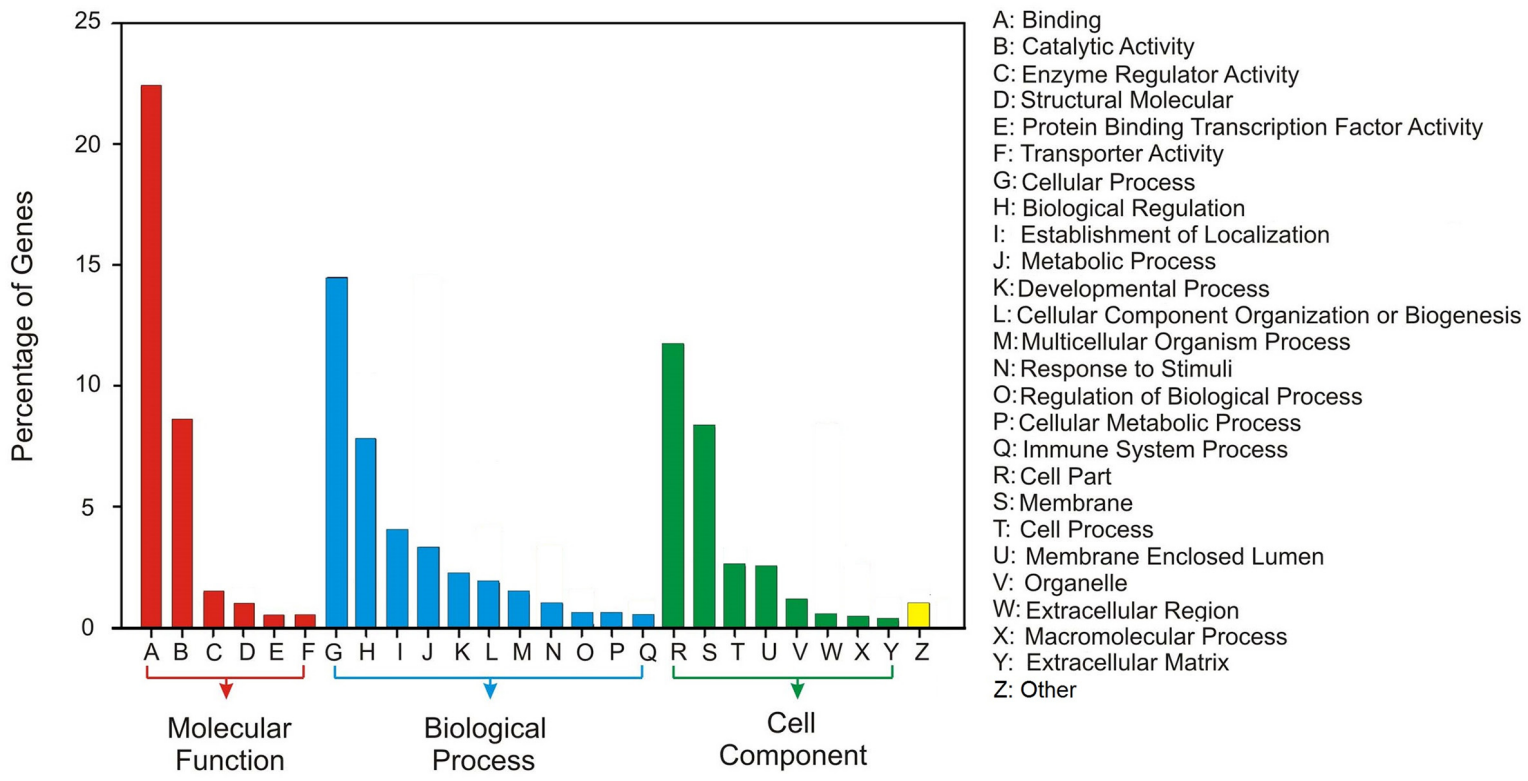
Occurrence of gene ontology terms among the gene clusters of *C*.*capillata* peri-rhopalial tissue transcriptome.

An analysis of the final annotated list (Fig 4) reveals a pattern of gene expression that, as defined by the Gene Ontology (GO) terms, is consistent with the nature of the tissue from which the RNA was isolated. As noted, the peri-rhopalial tissue is composed of a variety of cell types, including neurons, striated and non-striated (epitheliomuscular) muscle, cnidocytes and gland cells. Moreover, the peri-rhopalial tissue grows in size for the entire life of the animal so neurons, for example, are continually differentiating from precursors and elongating to populate the various nerve nets. The pattern of gene expression is what one might expect for a heterogeneous, metabolically-active and growing tissue. Importantly, examination of the results of the BLAST analysis of the fragment used for the assembly revealed many neurobiologically-relevant genes (e.g. ion channels, neurotransmitter receptors and transporters) that have the potential to provide great insight into the properties of MNN neurons and the evolution of the nervous system. From these observations we can conclude that our clustering approach does not only improve the numerical integrity of *de novo* assembled transcriptome, but also makes sense from the biological point of view, creating a better ground for meaningful observations and discoveries.

The final output contains only a subset of the entire transcriptome for which there is at least some clue regarding its function and evolutionary relationship to previously-studied proteins. This is the most informative part we need for the case study as well as many other research projects. The transcriptome of non-canonical model organisms often contains unique transcripts with unknown function that have no, or only distant, homologs in other genomes. An analysis of such genes is of scientific interest, but remains outside the scope of our study. The software we designed and the overall analysis workflow we propose is focused on reconstruction of the larger part of an unknown transcriptome, for which annotation by sequence similarity is possible.

The utility of our methods extends beyond just one species. For example, analysis of the rattlesnake (*Crotatus horridius*) transcriptome was a small part of the overall genome analysis and annotation for this species. The data includes a single run of Illumina Miseq (over 20 million reads). Initial assembly has been performed using Trinity and resulted 262,112 contigs. In expert opinion (personal communications) this number exceeds expectations for a reptile genome and likely to reflect low coverage and poor quality of RNA in the sample. After our super-scaffolding: 11,124 transcripts, 10,237 of them have annotated nearest homolog. Additional case studies are included in supplemental materials (S1 File).

In its present form, clusters of transcripts unite all kinds of fragments that sequence assembly failed to join on the basis of nucleotide sequence overlap. Clusters may include alternative splice forms and expressed forms of paralogous genes. For this reason the method we proposed for the cases of low-coverage transcriptome sequencing of non-canonical model organisms may not be justified for application in all RNAseq projects. For instance, denovo assembly a human RNAseq data (see S1 File) with Trinity results in 33,580 contigs. Additional processing by our method reduces this number to 23,116 for no apparent gain in functional annotation of reconstructed contigs. Future research and development may focus on the separation and detailed study of these expressed forms. However, for the intended application (characterization of transcriptome of non-model organisms) delineation of expression forms and closely related paralogous genes is excessive in details and would require extensive experimental validation.

### Implementation and availability

The pipeline has been developed using a combination of existing software and new code in C. The package contains three programs for clustering the raw transcript drafts, estimating expression values and determining transcript cluster statistics. Only the clustering application requires significant computational resources and can be supplemented with a scalable parallel version. Parallel code is implemented using POSIX standard threads and tested on Linux and CentOS machines (not available for Cygwin). A single-CPU version is also available. The open source software can be downloaded free of charge from the GitHub project: https://github.com/ptitsyn/clustering-rnaseq-without-reference-genome or acquired from the authors by request. The secondary deposit is also available at http://code.google.com/p/clustering-rnaseq-without-reference-genome/. This work is licensed under a Creative Commons Attribution 3.0 Unported License: http://creativecommons.org/licenses/by/3.0/

## Methods

### RNA preparation

Total RNA from 5 pieces of peri-rhopalial tissue was isolated with the Ambion® RNAqueous® Kit followed by an on-column DNA digestion using a Qiagen kit (cat. No. 79254). The quantity and quality of RNA was assessed on an Agilent BioAnalyser. Four micrograms of RNA with a RIN value of 8 was sent to SeqWrite, Inc. (Bellfort, Houston, TX) who prepared a cDNA library using the TruSeq kit from Illumina. The generated library was quality checked using the Agilent BioAnalyzer and sequenced on the MiSeq platform from Illumina.

### Workflow overview

**Fig 5:**
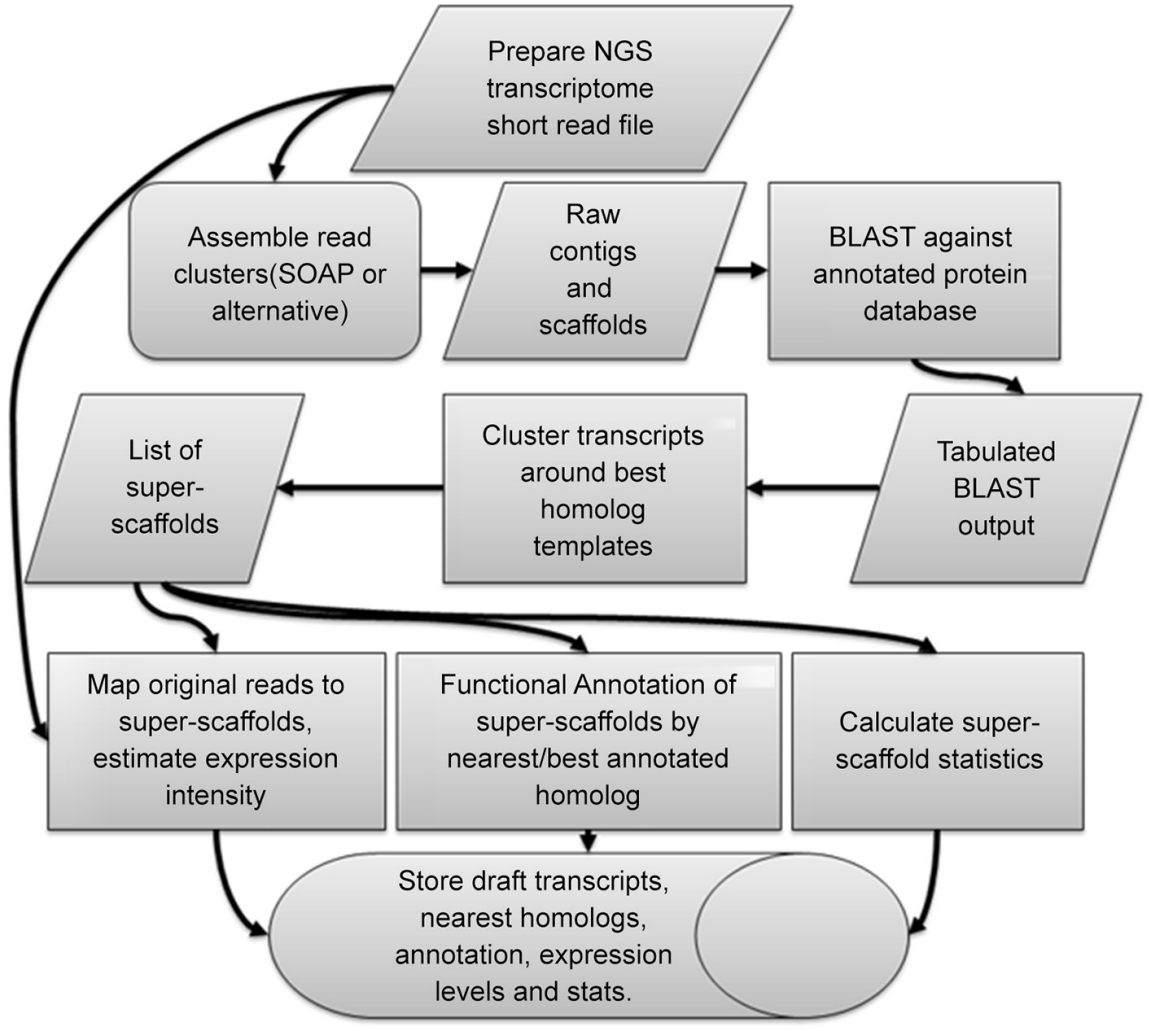
Overview of the analysis workflow.

The overview of computational workflow is given on Fig 5. For this case study we did not pipeline applications into a single script. This additional development can be done at the user's convenience when a study calls for automated application of the same software on a large number of data sets. In our case study we employed SOAPdenovo for the *de novo* assembly step. It is possible, with additional technical development, to substitute SOAPdenovo with different assembly software.

### Clustering algorithm

**Fig 6:**
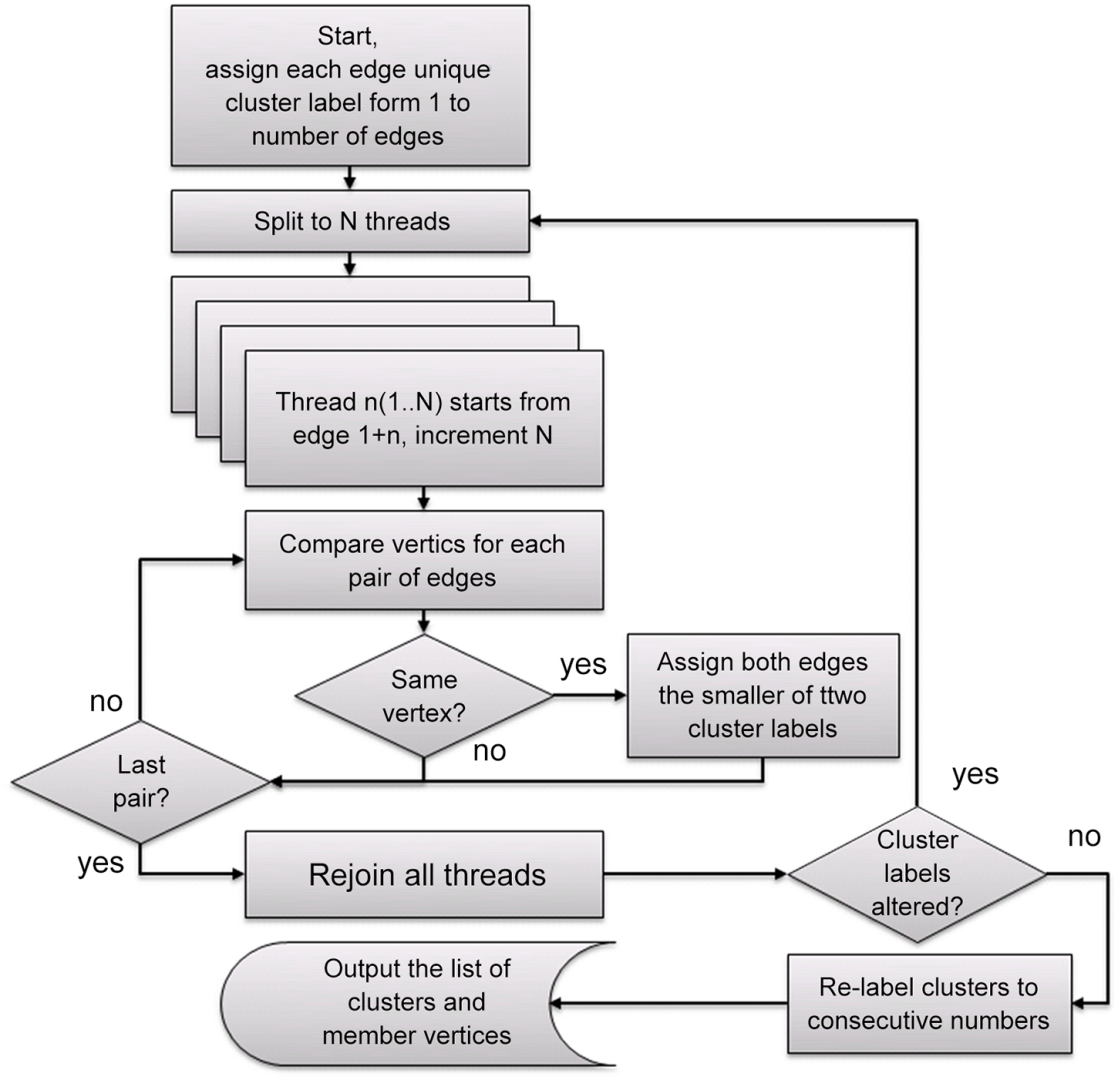
Clustering algorithm. The chart shows parallel versions of the iterative clustering algorithm that clusters raw scaffolds and contigs from *de novo* assembly (vertices) using information about their common homology to database proteins (edges) extracted from tabulated BLAST output.

The starting data for the algorithm is the list of edges. The tabulated output of the BLAST search used for input data is interpreted so that each BLAST hit is the edge of a graph connecting two vertexes—a query sequence and homologous database sequence. At the start, all edges are labeled with cluster numbers running from 1 to N (the number of edges in the input file). Foreach iteration, all edges on the list are compared to each other and pairs that share a vertex are given a cluster label that is the smaller of two original labels. For example, if edge (BLAST hit) number 45 shared a vertex (i.e. the query is homologous to the same database sequence) with edge 328, both edges will be labeled as cluster number 45. Iterations continue until no further cluster labels are updated. The resulting list is then sorted and re-labeled for continuous and consecutive order of clusters. As a result, all fragments that share a match to at least one database protein end up in one cluster. In addition, cluster statistics (such as longest and shortest edge, average edge length, number of members, etc.) are calculated and stored for further studies. Clustering is based on BLAST results, the e-value of a match can be used for estimation of the edge length. Smaller e-values indicate high similarity and higher degree of confidence in the reconstructed transcript cluster. In the case study we do not consider BLAST hits with e-value higher than 0.001. The flowchart of the algorithm is given on Fig 6. The input file is generated by a standard translated protein BLAST search of query (pre-assembled set of contigs and scaffolds) against the database of annotated sequences (such as SwissProt or UniProt database [21, 22]) with options for tabulated output and e-value cutoff (0.001 in our case study, but can be varied in different projects).

### Data for the case study

The data was received from the sequencing core facility (SeqWright, Inc., Houston, TX, see www.seqwright.com for more information) by secure FTP. The results of paired-end sequencing were in two files in FASTQ format each containing 8,557,893 reads. The length of a read was 150 nucleotides; the average insert size was 300 nucleotides.

## Supporting Information

S1 File Additional Case Studies. This is a zip archive that contains additional input data and results from other research projects involving our super-scaffolding method along with a brief description of the study and the files. The results may not reproduce exactly because the most current version of Swissprot database may include new homologous sequences.
(ZIP)

S1 Table Functional Annotation of clusters resulting from semantic superscaffolding. Inside is a table of functional annotation clusters exported from DAVID (Functional Annotation Clustering tool) [23]. Annotation is performed by proxy, i.e. using the nearest annotated homolog in SwissProt database to represent a cluster of transcripts generated by our software. The second tab of the same file contains the table of the clustered transcriptome in three columns: cluster name, nearest annotated homolog and read count.
(XLSX)
