# Supplementary material for: Semantic Assembly and Annotation of Draft RNAseq Transcripts without a Reference Genome
Source: PLoS One. 2015 Sep 22;10(9):e0138006. doi: 10.1371/journal.pone.0138006 (PMC4578894; doi:10.1371/journal.pone.0138006)
Supplement: S1 File — This is a zip archive that contains additional input data and results from other research projects involving our super-scaffolding method along with a brief description of the study and the files. The results may not reproduce exactly because the most current version of Swissprot database may include new homologous sequences. (ZIP) [file pone.0138006.s001.zip › Description of additional case studies.docx]

# Timber Rattlesnake (*Crotalus horridus*) transcriptome reconstruction

The sequence data has been obtained by courtesy of the owner, Dr. Doug Rhoads (http://www.uark.edu/ua/drhoads/) and MCBIOS Collaborative Effort described in:

<ftp://ftp.erc.msstate.edu/outgoing/wss2/Sanders_MCBIOS_Rattlesnake_2015.pdf>

Our analysis started with raw reads downloaded from the project website. The data includes a single run of Illumina Miseq (over 20 million reads). Initial assembly has been performed using Trinity and resulted **262,112** contigs. After our super-scaffolding: **11,124** transcripts, **10,237** of them have annotated nearest homolog.

The processed data files include:

rs_tr.fa – Reconstruction of transcriptome by Trinity in FASTA-format. This data is used as input for our super-scaffolding.

rs_table.txt – list of resulting superscaffolds with homologs in SwissProt

rs_anno.xlsx – the final resulting table of annotated transcripts

# Human RNAseq

The data is part of the 1000 genomes project, sample HG00100 downloaded from

<http://www.ebi.ac.uk/arrayexpress/experiments/E-GEUV-1/samples/>

There is no reason to apply our super-scaffolding method to this data except for benchmarking. Trinity assembly produced 33580 contigs. Application of super-scaffolding procedure reduced the number of reconstructed transcripts to 23116.

hs_tr.fa: Trinity reconstruction of transcriptome in FASTA format

hs_tr_sscaf.fa: results of super-scaffolding in FASTA format
